# The Use of 3D Printed Models for Surgical Simulation of Cranioplasty in Craniosynostosis as Training and Education

**DOI:** 10.3390/brainsci13060894

**Published:** 2023-06-01

**Authors:** Jean Francois Uhl, Albert Sufianov, Camillo Ruiz, Yuri Iakimov, Huerta Jose Mogorron, Manuel Encarnacion Ramirez, Guillermo Prat, Barbara Lorea, Matias Baldoncini, Evgeniy Goncharov, Issael Ramirez, José Rafael Cerda Céspedes, Renat Nurmukhametov, Nicola Montemurro

**Affiliations:** 1 Anatomy Department, Paris University and UNESCO Chair of Digital Anatomy, 75100 Paris, France; 2 Federal Center of Neurosurgery, Sechenov University, 119435 Moscow, Russia; 3 Laboratorio de Investigaciones Morfológicas Aplicadas, Universidad Nacional de La Plata, La Plata B1900, Argentina; 4 Neurological Surgery, Peoples Friendship University of Russia, 103274 Moscow, Russia; 5 Laboratory of Microsurgical Neuroanatomy, Second Chair of Gross Anatomy, School of Medicine, University of Buenos Aires, Buenos Aires B1406, Argentina; 6 Traumatology and Orthopedics Center, Central Clinical Hospital of the Russian Academy of Sciences, 103272 Moscow, Russia; 7 Neurosurgery Department, The Royal Melbourne Hospital, Melbourne 3000, Australia; 8 Department of Surgery, Universidad Tecnológica de Santiago (UTESA), Santiago 5100, Dominican Republic; 9 Department of Neurosurgery, Azienda Ospedaliero Universitaria Pisana (AOUP), University of Pisa, 56100 Pisa, Italy

**Keywords:** 3D printing, craniosynostosis, cranioplasty, 3D modeling, 3D printed model, simulation, surgical education, neurosurgery

## Abstract

Background: The advance in imaging techniques is useful for 3D models and printing leading to a real revolution in many surgical specialties, in particular, neurosurgery. Methods: We report on a clinical study on the use of 3D printed models to perform cranioplasty in patients with craniosynostosis. The participants were recruited from various medical institutions and were divided into two groups: Group A (*n* = 5) received traditional surgical education (including cadaveric specimens) but without using 3D printed models, while Group B (*n* = 5) received training using 3D printed models. Results: Group B surgeons had the opportunity to plan different techniques and to simulate the cranioplasty. Group B surgeons reported that models provided a realistic and controlled environment for practicing surgical techniques, allowed for repetitive practice, and helped in visualizing the anatomy and pathology of craniosynostosis. Conclusion: 3D printed models can provide a realistic and controlled environment for neurosurgeons to develop their surgical skills in a safe and efficient manner. The ability to practice on 3D printed models before performing the actual surgery on patients may potentially improve the surgeons’ confidence and competence in performing complex craniosynostosis surgeries.

## 1. Introduction

The use of three-dimensional (3D) models of the human body led to a revolution in the field of anatomy and surgery, especially in neurosurgery. Its usefulness in teaching anatomy, as well as in all phases of surgical training, is well known [1,2]. Furthermore, it can overcome, without replacing it for the moment, the difficulty of obtaining human bodies for anatomical dissection. 

3D models allow simulation of the surgery by planning it with an effective model, which subsequently aids the surgeon while operating on the real patient. Although there are many ways to make a 3D anatomical model, the best sources are anatomical slices taken by computer tomography (CT) or magnetic resonance imaging (MRI) [2,3,4].

For pedagogical matters, the models obtained from the Visible Human Project data provide an interesting source to produce 3D models. DICOM data (Digital Imaging and Communication in Medicine) obtained from medical imaging can be used as a raw source for 3D modeling. These new technologies should be combined with cadaveric dissection, which offers an experience that cannot be replaced [3].

There are different types of 3D printers. For this study, Fused Deposition Modeling (FDM) was used. This is an economical and easy-to-learn technique [4] that offers models suitable for simulation. The material used to print is plastic (PLA) because its hardness is similar to bone. Most of the reviews available on the clinical use of 3D printing [5,6,7] agree that the main areas of application are the creation of implants, anatomical models (implant shaping and surgical planning), molds for prosthetics, surgical guides, and pedagogical applications. Neurosurgery benefits from 3D printing in several ways [8,9,10], as 3D printed models allow the possibility of getting tactile feedback and help in recognizing anatomical patterns in the preoperative stage.

Craniosynostosis is a condition in which one or more of the fibrous sutures in a young infant’s skull prematurely fuses by turning into bone (ossification), thereby changing the growth pattern of the skull [11]. Because the skull cannot expand perpendicular to the fused suture, it compensates by growing in the direction parallel to the closed sutures. Sometimes the resulting growth pattern provides the necessary space for the growing brain, but results in an abnormal head shape and abnormal facial features [11]. The primary goal of surgical intervention is to allow normal cranial vault development to occur [12,13,14,15]. This can be achieved by excision of the prematurely fused suture and correction of the associated skull deformities [11].

In this article, we aim to review the current literature on the use of 3D printed models for surgical simulation of cranioplasty in craniosynostosis as a tool for training and education. Overall, the utilization of 3D printed models in surgical simulation for cranioplasty in craniosynostosis holds promise as a valuable tool in enhancing surgical skills and improving patient outcomes. Proper utilization of 3D printing technology in surgical training can contribute to improved surgical outcomes and patient care in the management of craniosynostosis. The purpose of this paper is to assess the usefulness of 3D printed models to improve the learning curve of cranioplasty in craniosynostosis in neurosurgeons.

## 2. Methods

### 2.1. Study Design

This study utilized a descriptive research design to explore the use of 3D printed models for surgical simulation of cranioplasty in craniosynostosis as a training and educational tool. The study included a convenience sample of 10 neurosurgeons. Participants were recruited from various medical institutions and had less than 5 years of surgical experience. Participants were 9 male and 1 female, whereas the mean age was 30.4 years old. Participants finished their residency between 2 and 3 years ago, declared to have performed less than 3 cranioplasties (mean 1.7 procedures) in craniosynostosis, and were selected based on their availability and willingness to participate in the study.

### 2.2. D Printing and Model Creation

Preoperative CT scans of patients diagnosed with craniosynostosis were used to create 3D printed models. The CT scan data was processed using specialized software to generate a digital 3D model of the patient’s skull. The digital model was then 3D printed using a biocompatible material to create a physical replica of the patient’s skull. The models were designed to replicate the anatomy of the cranial defect in craniosynostosis patients, including the shape, size, and location of the defect as well as any associated anatomical landmarks.

### 2.3. Surgical Simulation

3D printed models were used for surgical simulation of cranioplasty procedures in craniosynostosis. Participants were divided into two groups: Group A (*n* = 5) received traditional surgical education (cadaveric specimens) without 3D printed models, while Group B (*n* = 5) received training using 3D printed models. Participants in Group B were able to practice various surgical techniques, such as bone reshaping and fixation, on 3D printed models before performing the actual surgery on patients.

### 2.4. Assessment of Learning Outcomes

The learning outcomes of the participants were assessed through objective measurements, including the accuracy of bone reshaping and fixation and overall performance scores evaluated by experienced craniofacial surgeons. Participant feedback and subjective evaluations of 3D printed models as a training and educational tool were also collected.

### 2.5. Data Analysis

Descriptive statistics were used to summarize the demographic characteristics of the participants. Comparative analysis was conducted to assess the differences in learning outcomes between Group A and Group B. Qualitative data from participant feedback and subjective evaluations were analyzed thematically to identify common themes and patterns.

### 2.6. Ethical Considerations

This study was conducted in accordance with the ethical guidelines and principles outlined by the Declaration of Helsinki. Institutional Review Board (IRB) approval was obtained prior to study initiation. Informed consent was obtained from all participants and their confidentiality and privacy were preserved.

### 2.7. Description of the Case

**Figure 1:**
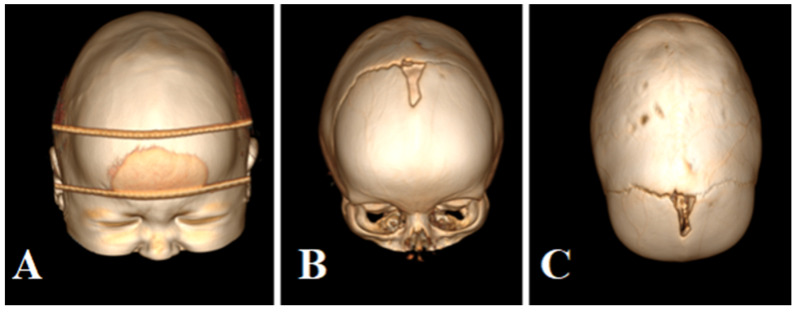
Patient’s CT scan before the surgery. (**A**) Frontal view with head tape fixation for CT examination, (**B**) frontal view with skull reconstruction, (**C**) top view with skull reconstruction.

The patient is 1 year old, without any developmental delays diagnosed with sagittal craniosynostosis confirmed by CT scan shown in Figure 1.

### 2.8. Procedure to Build and Print the 3D Model

**Figure 2:**
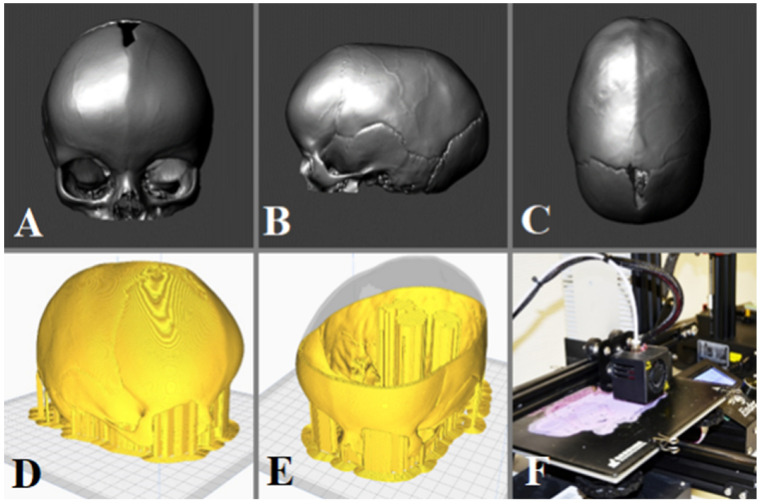
Model of the patient after preparing and correcting artifacts with Meshmixer^®^ (from Autodesk: San Rafael, CA, USA). (**A**) Frontal, (**B**) lateral, and (**C**) top view with skull reconstruction. (**D**,**E**) Building of slices of the cranium and support with a Cura^®^. (**F**) Fuse Filament Fabrication (FFF) 3D Printer.

The data was obtained from a CT scan using the Canon Aquilion One. The DICOM data obtained was analyzed using Horos^®^, free software [16] that when based on the Hounsfield Unit threshold (cortical bone has a high value), allows the creation of a 3D vectorial model. This mesh has high accuracy, but it may still have some artifacts that can be exported in obj format and then refined and improved by using Meshmixer^®^ 3.5 (Oakland, CA, USA) [17] from Autodesk (Figure 2A–C). The final 3D model was imported into Cura^®^ 4.6 software (Ultimaker, The Netherlands) [18] where the printing parameters were defined and exported in “.gcode” format so that the FFF 3D printer (Anet A8^®^, Anet Technology Co., Shenzhen, China) could read it and deposit the fused material as instructed to create the skulls (Figure 2D–F).

**Figure 3:**
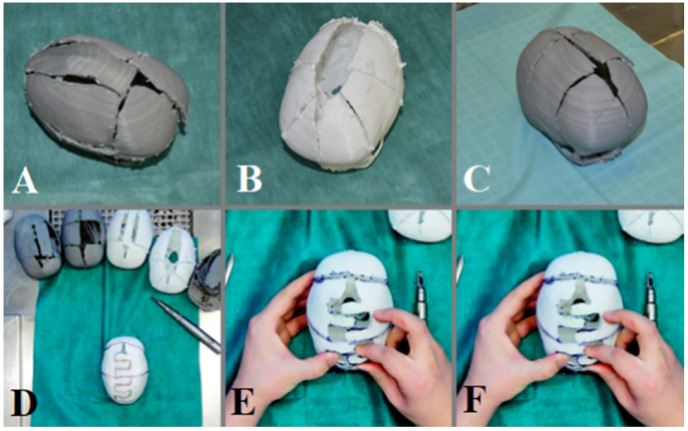
Simulation of the surgeon’s own technique on a 3D printed skull model. Participants trained with 6 printed models and realized their own variant of cranioplasty. (**A**) free flap skull reconstruction, (**B**) semi-free flaps skull reconstruction, (**C**) semi-free flaps parietal skull reconstruction. (**D**) Drawing of the skull incisions, (**E**) performance of the planned bone sections, (**F**) fixation of the bones with sutures.

Following the introductory session, the participants engaged in a hands-on practice session with the 3D printed models. Each participant in Group B had the opportunity to perform simulated cranioplasty procedures on the models, guided by experienced instructors. This hands-on practice allowed the neurosurgeons to gain practical experience in utilizing 3D printed models, familiarize themselves with the surgical techniques, and develop their skills in a controlled and realistic environment (Figure 3).

After the hands-on practice session, a feedback and debriefing session was conducted to address any questions, concerns, or challenges that the participants encountered during the training. This session provided an opportunity for instructors to provide constructive feedback, clarify any misconceptions, and further reinforce the key concepts and techniques related to the use of 3D printed models in cranioplasty for craniosynostosis. The feedback and debriefing session aimed to enhance the participants’ understanding and performance and to facilitate continuous learning and improvement.

Overall, the training program for the use of 3D printed models for surgical simulation of cranioplasty in craniosynostosis included an introductory session, a hands-on practice session, and a feedback and debriefing session (Table 1 and Table 2). Table 3 shows survey questions on the involvement and satisfaction of the participants.

## 3. Results

### 3.1. Demographic Characteristics

The study included a convenience sample of 10 neurosurgeons with less than 5 years of surgical experience. The participants were recruited from various medical institutions and were divided into two groups: Group A (*n* = 5) received traditional surgical education (including cadaveric specimens) but without training with 3D printed models, while Group B (*n* = 5) received training using 3D printed models.

### 3.2. Learning Outcomes

The learning outcomes of the participants were assessed through objective measurements and qualitative feedback (Table 4). In addition, objective measurements included the accuracy of bone reshaping and fixation as well as overall performance scores evaluated by experienced craniofacial surgeons. Results showed that participants in Group B, who received training using 3D printed models, demonstrated better accuracy in bone reshaping, fixation, and less time in performing procedures compared to Group A, who received traditional surgical education using cadaveric specimens.

The overall performance scores of Group B were also higher compared to Group A, indicating that the use of 3D printed models for surgical simulation of cranioplasty in craniosynostosis was effective in improving surgical skills. Qualitative feedback from participants in Group B also indicated that 3D printed models were a valuable training and educational tool (Table 4). Participants reported that models provided a realistic and controlled environment for practicing surgical techniques, allowed for repetitive practice, and helped in visualizing the anatomy and pathology of craniosynostosis. Models were praised for their accuracy in replicating the cranial defect and associated anatomical landmarks, which enhanced the learning experience. It is important to note that this study represents an initial approach, focusing on a small group of individuals, that highlights the potential benefits of 3D printing in medical applications. However, it is hypothesized that this enhanced comfort may also lead to reduced surgical time. To validate this hypothesis, further comparative studies incorporating different variables need to be conducted.

### 3.3. Themes and Patterns

Thematic analysis of qualitative data from participant feedback and subjective evaluations identified several common themes and patterns. These included the benefits of using 3D printed models for surgical simulation, such as improved understanding of the anatomy and pathology of craniosynostosis, enhanced spatial perception and depth perception, increased confidence in performing the surgery, and reduced risk of complications in real patients. Participants also reported that the use of 3D printed models allowed for repetitive practice, which helped improve their surgical skills and confidence. The accuracy and realism of the models were highly appreciated, as they closely replicated the anatomy and pathology of craniosynostosis, allowing for a realistic and immersive training experience. This study suggests that the use of 3D printed models for surgical simulation of cranioplasty in craniosynostosis is an effective training and educational tool for neurosurgeons. These models provide a realistic and controlled environment for practicing surgical techniques, improve accuracy in bone reshaping and fixation, enhance spatial perception and depth perception, and increase confidence in performing the surgery. The accuracy and realism of the models also contribute to a valuable learning experience. Further research and larger-scale studies may be warranted to confirm these findings and explore the long-term impact of using 3D printed models in surgical education and training for craniosynostosis. Overall, the use of 3D printed models for surgical simulation of cranioplasty in craniosynostosis has the potential to improve surgical skills and patient outcomes in neurosurgical practice. Further research and larger-scale studies may be warranted to confirm these findings and explore the long-term impact of using 3D printed models in surgical education and training for craniosynostosis.

Also, we found that it reduced blood loss and time of surgery while lowering the risk of infection. In addition, this method is interesting for young neurosurgeons. It presents a unique opportunity to step towards new types of operations that they had not conducted before without risk to the patient.

## 4. Discussion

Surgeons benefit from the cadaveric-dissection process [8], as it helps them to learn regional anatomy and become familiar with the instrumental material and manual skills required. However, not all training centers can easily acquire corpses, given the associated costs and legal implications. Cadaver treatment for training and storage also requires a highly specialized center. The difficulty of obtaining and maintaining corpses has been considered a major challenge, particularly in low-income countries or in non-university hospital centers.

During the COVID-19 pandemic, cadaveric practices have been significantly reduced, demonstrating that, in the face of adversity, human specimen practice can be significantly impacted [19,20]. Simulation with 3D printing allows the creation of several models from one patient and allows one to reproduce this process as many times as needed for training. While 3D printing has many advantages, there are some limitations of this process. The materials available are limited by their thermodynamic characteristics. Complex and large-scale models may result in deficient printing, as they are time-consuming and difficult to print, among others [21]. Rapid prototyping allows for the quick manufacture of 3D models from medical imaging data, giving the surgeon the possibility not only of visual 2D information but also tactile feedback from the 3D model [22]. It also allows training for the surgery in order to plan the best surgical approach.

Fused filament fabrication (FFF) 3D printing is available and widespread. PLA (polylactic acid) has many advantages, such as non-toxicity, and is made by the polymerization of lactic acid or lactide. It is, therefore, not an oil derivative, but is produced by the bacterial fermentation of carbohydrates (corn, carp, cassava). The sterilization process is carried out with low-temperature sterilization and 100% ethylene oxide is the standard protocol since it does not affect the physical or anatomical properties of the material [23]. At the same time, the use of PLA has its drawbacks since it has a low temperature of crystallization (55 °C) and melting (180 °C). When performing drilling, it can melt, so you need to use a low-speed drill. 

One of the key findings of this study is that the use of 3D printed models for surgical simulation in craniosynostosis appears to be a promising tool for the training and education of young neurosurgeons. The hands-on practice session with 3D printed models allowed the participants to gain practical experience in performing simulated cranioplasty procedures, including bone reshaping and fixation. 

The participants’ feedback and subjective evaluations of 3D printed models were generally positive, with many participants reporting the high accuracy and anatomical similarity of the models to actual craniosynostosis patients. This suggests that 3D printed models were able to accurately replicate the anatomy of the cranial defect and associated landmarks, which is crucial for surgical simulation purposes. The participants also reported that 3D printed models were helpful in improving their understanding of the surgical techniques and in enhancing their spatial perception and hand-eye coordination. This supports the potential of 3D printed models as an effective educational tool for neurosurgeons to learn and practice complex surgical procedures.

Another important finding of this study is that the use of 3D printed models may have advantages over traditional surgical education using cadaveric specimens. While cadaveric specimens have been widely used for surgical training, they have limitations such as limited availability, cost, and ethical considerations. In contrast, 3D printed models can be easily replicated and customized based on patient-specific CT scan data, making them more accessible and cost-effective [24,25,26,27]. Additionally, 3D printed models do not pose ethical concerns and do not have the potential risk of transmitting infections. This suggests that 3D printed models, in association with or without telemedicine, may be a viable alternative or complement to traditional cadaveric specimens for surgical simulation in craniosynostosis [28,29,30,31,32].

However, there are also some limitations of using 3D printed models for surgical simulation in craniosynostosis that should be considered. One limitation is that the accuracy and quality of 3D printed models depend on the quality of the CT scan data and the processing software used. Artifacts or inaccuracies in the CT scan data or the 3D modeling process may affect the accuracy and realism of the 3D printed model. Another limitation is that while 3D printed models can provide a realistic representation of the anatomy and pathology of craniosynostosis, they may not fully replicate the tactile feedback and sensation of performing surgery on actual patients. Haptic feedback and tactile sensation are important aspects of surgical training, and the absence of these elements in 3D printed models may limit their effectiveness in fully simulating real surgical procedures.

### Limitations of the Study

The main limitation of this study is the small sample of neurosurgeons who were trained and interviewed. Another limitation is that all neurosurgeons reflect a single-center experience, where they have been trained during residency. Further studies should be conducted in an international multicenter setting with a large sample of neurosurgeons to evaluate the effective utility of these 3D printed models.

## 5. Conclusions

3D models have many applications in surgery. Our interest was not only to train young surgeons but to tailor treatment to each patient. Surgical simulation with 3D modeling and printing is useful in the planning of surgery, particularly in neurosurgery. In the future, we believe these new tools will improve the technique of surgeons. Finally, they also constitute a fantastic educational and training tool for young surgeons and potentially can lower complication rates. The use of 3D printed models can provide a realistic and controlled environment for neurosurgeons to develop their surgical skills in a safe and efficient manner. The ability to practice on 3D printed models before performing the actual surgery on patients may potentially improve the surgeons’ confidence and competence in performing complex craniosynostosis surgeries.

## Figures and Tables

**Table 1:** Stage of the training program and its description.

Stage of Training Program	Description
Introductory Session	An initial session introducing the participants to the use of 3D printed models for surgical simulation of cranioplasty in craniosynostosis. This session may cover topics such as the benefits of using 3D printed models, the anatomy and pathology of craniosynostosis, and the importance of surgical simulation in improving surgical skills.
Hands-on Practice Session	A hands-on practice session where the participants get to work with 3D printed models, simulating cranioplasty procedures. This may involve performing various surgical steps such as planning the incisions, drilling, and fixation of the bone segments using 3D printed models. Participants may receive guidance and feedback from experienced instructors to refine their skills.
Feedback and Debriefing Session	A session where participants can ask questions, clarify doubts, and receive feedback on their performance during the hands-on practice session. This session may also include debriefing on the surgical simulation exercise, discussing the challenges faced, and identifying areas for improvement. Participants may receive constructive feedback and suggestions for further improvement in their surgical skills using 3D printed models.

**Table 2:** This table shows the advantages and disadvantages of training with (Group B) or without (Group A) 3D Model.

Aspect	Surgeons without 3D Model Training (Group A)	Surgeons with 3D Model Training (Group B)
Number of Surgeons	5	5
Training Program	N/A	Comprehensive training program
Introduction Session	N/A	Included
Hands-on Practice with 3D Models	N/A	Included
Feedback and Debriefing Session	N/A	Included
Experience with 3D Printed Models	No exposure	Exposure to realistic models
Surgical Simulation	N/A	Realistic surgical simulation
Surgical Education	Standard understanding	Enhanced understanding
Skill Development	Standard surgical skills	Improved surgical skills
Confidence in Cranioplasty	Standard confidence	Increased confidence
Complication Rates	Standard complication rates	Potentially reduced
Patient Outcomes	Standard patient outcomes	Potentially improved

N/A indicates that the aspect was not applicable to surgeons without 3D model training as they did not receive any training related to 3D printed models for cranioplasty in craniosynostosis. The comparative table highlights the potential benefits of the training program with 3D printed models, including improved surgical skills, increased confidence, potentially reduced complication rates, and potentially improved patient outcomes, as compared to surgeons without such training.

**Table 3:** Participant engagement and satisfaction survey.

Question	Answers
Rate your overall engagement during the educational session	1—Not engaged at all 2—Not engaged 3—Barely engaged 4—Moderate engaged 5—Highly engaged
How would you describe your level of interest in the educational content?	1—Very low 2—Low 3—Moderate 4—High 5—Very high
Did the 3D model enhance your understanding and retention of the subject matter?	1—Yes, significantly 2—Yes, to some extent 3—No, not really 4—No, not at all
How would you rate the visual appeal and clarity of the 3D model?	1—Poor 2—Fair 3—Good 4—Very good 5—Excellent
Compared to traditional/classical methods, do you believe the 3D model helped to make the educational content more engaging?	1—Yes, significantly more engaging 2—Yes, somewhat more engaging 3—No, about the same level of engagement 4—No, less engaging
Did the 3D model improve your understanding of the subject matter compared to the classical method?	1—Yes, significantly 2—Yes, to some extent 3—No, not really 4—No, not at all
How confident do you feel in applying the knowledge learned from the 3D model to real-life scenarios?	1—Very confident 2—Confident 3—Somewhat confident 4—Not confident at all
Did the 3D model facilitate your ability to grasp complex concepts and relationships?	1—Yes, significantly 2—Yes, to some extent 3—No, not really 4—No, not at all
Rate your improvement in knowledge and skills after the educational session	1—No improvement 2—Slight improvement 3—Improvement 4—Moderate improvement 5—Significant improvement
Did the 3D model provide a better learning experience compared to the classical method?	1—Yes, significantly better 2—Yes, somewhat better 3—No, about the same learning experience 4—No, worse

**Table 4:** Satisfaction survey and learning outcomes.

Question	Participating Surgeons of the Group B				
	1	2	3	4	5
Rate your overall engagement during the educational session	Highly engaged	Highly engaged	Moderate engagement	Highly engaged	Highly engaged
How would you describe your level of interest in the educational content?	High	Very high	Moderate	High	Very high
Did the 3D model enhance your understanding and retention of the subject matter?	Yes, significantly	Yes, to some extent	Yes, to some extent	Yes, to some extent	Yes, significantly
How would you rate the visual appeal and clarity of the 3D model?	Good	Very good	Good	Good	Excellent
Compared to traditional/classical methods, do you believe the 3D model helped to make the educational content more engaging?	Yes, significantly more engaging	Yes, somewhat more engaging	Yes, somewhat more engaging	Yes, somewhat more engaging	Yes, somewhat more engaging
Did the 3D model improve your understanding of the subject matter compared to the classical method?	Yes, to some extent	Yes, significantly	Yes, significantly	Yes, significantly	Yes, significantly
How confident do you feel in applying the knowledge learned from the 3D model to real-life scenarios?	Confident	Very confident	Very confident	Very confident	Very confident
Did the 3D model facilitate your ability to grasp complex concepts and relationships?	Yes, significantly	Yes, to some extent	Yes, significantly	Yes, significantly	Yes, significantly
Rate your improvement in knowledge and skills after the educational session	Significant improvement	Significant improvement	Significant improvement	Significant improvement	Significant improvement
Did the 3D model provide a better learning experience compared to the classical method?	Yes, somewhat better	Yes, significantly better	Yes, significantly better	Yes, significantly better	Yes, significantly better

## References

[1] Condino S., Montemurro N., Cattari N., D’amato R., Thomale U., Ferrari V., Cutolo F. (2021). Evaluation of a Wearable AR Platform for Guiding Complex Craniotomies in Neurosurgery. Ann. Biomed. Eng..

[2] Montemurro N., Condino S., Cattari N., D’amato R., Ferrari V., Cutolo F. (2021). Augmented Reality-Assisted Craniotomy for Parasagittal and Convexity En Plaque Meningiomas and Custom-Made Cranio-Plasty: A Preliminary Laboratory Report. Int. J. Environ. Res. Public Health.

[3] de Notaris M., Topczewski T., de Angelis M., Ensenat J., Alobid I., Gondolbleu A.M. (2013). Anatomic skull base education using advanced neuroimaging 0g techniques. World Neurosurg..

[4] Baskaran V., Štrkalj G., Štrkalj M., Di Ieva A. (2016). Current Applications and Future Perspectives of the Use of 3D Printing in Anatomical Training and Neurosurgery. Front. Neuroanat..

[5] Tack P., Victor J., Gemmel P., Annemans L. (2016). 3D-printing techniques in a medical setting: A systematic literature review. Biomed. Eng. Online.

[6] Rengier F., Mehndiratta A., von Tengg-Kobligk H., Zechmann C.M., Unterhinninghofen R., Kauczor H.-U., Giesel F.L. (2010). 3D printing based on imaging data: Review of medical applications. Int. J. Comput. Assist. Radiol. Surg..

[7] Ganguli A., Pagan-Diaz G.J., Grant L., Cvetkovic C., Bramlet M., Vozenilek J., Kesavadas T., Bashir R. (2018). 3D printing for preoperative planning and surgical training: A review. Biomed. Microdevices.

[8] Ian G., David W., Rosen D.W., Stucker B. (2014). Extrusion-Based Systems. Additive Manufacturing Technologies.

[9] Mishra R., Narayanan M.K., Umana G.E., Montemurro N., Chaurasia B., Deora H. (2022). Virtual Reality in Neurosurgery: Beyond Neurosurgical Planning. Int. J. Environ. Res. Public Health.

[10] Montemurro N., Condino S., Carbone M., Cattari N., D’amato R., Cutolo F., Ferrari V. (2022). Brain Tumor and Augmented Reality: New Technologies for the Future. Int. J. Environ. Res. Public Health.

[11] Slater B.J., Lenton K.A., Kwan M.D., Gupta D.M., Wan D.C., Longaker M.T. (2008). Cranial sutures: A brief review. Plast. Reconstr. Surg..

[12] Panchal J., Uttchin V. (2003). Management of craniosynostosis. Plast. Reconstr. Surg..

[13] Sanan A., Haines S.J. (1997). Repairing holes in the head: A history of cranioplasty. Neurosurgery.

[14] Durand J.L., Renier D., Marchac D. (1997). L’histoire des cranioplasties [The history of cranioplasty]. Ann. Chir. Plast. Esthet..

[15] Shah A.M., Jung H., Skirboll S. (2014). Materials used in cranioplasty: A history and analysis. Neurosurg. Focus.

[16] Horos. https://fr.freedownloadmanager.org/Mac-OS/Horos-GRATUIT.html.

[17] Meshmixer 3.5.474. https://meshmixer.updatestar.com/fr.

[18] Cura 3.6.0. https://cura.updatestar.com/fr.

[19] Ramirez M.E., Pena I.R., Castillo R.E.B., Sufianov A., Goncharov E., Sanchez J.A.S., Colome-Hidalgo M., Nurmukhametov R., Céspedes J.R.C., Montemurro N. (2023). Development of a 3D Printed Brain Model with Vasculature for Neurosurgical Procedure Visualisation and Training. Biomedicines.

[20] Ramirez M.D.J.E., Nurmukhametov R., Musa G., Castillo R.E.B., Encarnacion V.L.A., Sanchez J.A.S., Vazquez C.A., Efe I.E. (2022). Three-Dimensional Plastic Modeling on Bone Frames for Cost-Effective Neuroanatomy Teaching. Cureus.

[21] Wang X., Jiang M., Zhou Z.W., Gou J.H., Hui D. (2017). 3D printing of polymer matrix composites: A review and prospective. Compos. Part B Eng..

[22] Petzold R., Zeilhofer H.F., Kalender W.A. (1999). Rapid protyping technology in medicine--basics and applications. Comput. Med. Imaging Graph..

[23] Ramirez M.D.J.E., Nurmukhametov R., Bernard E., Peralta I., Efe I.E. (2022). A Low-Cost Three-Dimensional Printed Retractor for Transforaminal Lumbar Interbody Fusion. Cureus.

[24] Kuo J.R., Wang C.C., Chio C.C., Cheng T.J. (2004). Neurological improvement after cranioplasty-analysis by transcranial doppler ultrasonography. J. Clin. Neurosci..

[25] Manfiotto M., Mottolese C., Szathmari A., Beuriat P.-A., Klein O., Vinchon M., Gimbert E., Roujeau T., Scavarda D., Zerah M. (2017). Decompressive craniectomy and CSF disorders in children. Childs Nerv. Syst..

[26] Honeybul S., Janzen C., Kruger K., Ho K.M. (2016). The Incidence of Neurologic Susceptibility to a Skull Defect. World Neurosurg..

[27] Golz T., Graham C.R., Busch L.C., Wulf J., Winder R.J. (2010). Temperature elevation during simulated polymethylmethacrylate (PMMA) cranioplasty in a cadaver model. J. Clin. Neurosci..

[28] Blum K.S., Schneider S.J., Rosenthal A.D. (1997). Methyl methacrylate cranioplasty in children: Long-term results. Pediatr. Neurosurg..

[29] Marchac D., Greensmith A. (2008). Long-term experience with methylmethacrylate cranioplasty in craniofacial surgery. J. Plast. Reconstr. Aesthet. Surg..

[30] Montemurro N. (2022). Telemedicine: Could it represent a new problem for spine surgeons to solve?. Glob. Spine J..

[31] Grant G.A., Jolley M., Ellenbogen R.G., Roberts T.S., Gruss J.R., Loeser J.D. (2004). Failure of autologous bone-assisted cranioplasty following decompressive craniectomy in children and adolescents. J. Neurosurg..

[32] van de Vijfeijken S.E., Münker T.J., Spijker R., Karssemakers L.H., Vandertop W.P., Becking A.G., Ubbink D.T., Dubois L., Milstein D., Depauw P. (2018). Autologous Bone Is Inferior to Alloplastic Cranioplasties: Safety of Autograft and Allograft Materials for Cranioplasties, a Systematic Review. World Neurosurg..

